# COVID-19, mathematics education, and the evaluation of expert knowledge

**DOI:** 10.1007/s10649-021-10097-2

**Published:** 2021-08-27

**Authors:** David Kollosche, Wolfram Meyerhöfer

**Affiliations:** 1 University of Klagenfurt, Klagenfurt, Austria; 2 Schulzentrum Am Stern, Potsdam, Germany

**Keywords:** Civic education, Mathemacy, Mathematical literacy, Reflection on mathematics, Expert-layperson communication, COVID-19

## Abstract

Maturity and citizenship in a democracy require that laypersons are able to critically evaluate experts’ use of mathematics. Learning to critically reflect on the use of mathematics, including the acquisition of the mathematical knowledge and skills required to that end, has been repeatedly postulated as an indispensable goal of compulsory education in mathematics. However, it remained unclear in how far such reflection is possible, even for the well-educated layperson in mathematics. We use different discussions in German mass media on the pandemic policy in the SARS-CoV-2 crisis in 2020 as examples with far-reaching individual and social consequences. The selected discussions build heavily on mathematical concepts such as mortality rates, casualty numbers, reproduction numbers, and exponential growth. We identify the concepts and discuss how far they can be understood by laypersons. On the one hand, we found that some mathematical models are inappropriate, which can also be determined by laypersons. On the other hand, we found uses of mathematics where ideal concepts are intermingled with complex statistical concepts. While only the ideal concepts can be understood by laypersons, only the statistical concepts lead to actual data. The identification of both types of concepts leads to a situation where the use of mathematics evades social control and opens spaces for misconceptions and manipulation. We conclude that the evaluation of experts’ use of mathematics by laypersons is not possible in all relevant cases, and we discuss possible implications of this result.

## Mathematics in the pandemic

In 2020, the world experienced a global crisis as many countries invoked extreme political measures in reaction to the spread of the coronavirus disease 2019 (COVID-19), caused by the “severe acute respiratory syndrome coronavirus 2” (SARS-CoV-2), which is publicly also referred to as “the coronavirus”. The shock caused by media reports of overburdened medical facilities and human casualties associated to the virus as well as warnings by virologists, epidemiologists, and other experts led a majority of countries worldwide to react forcefully with a social lockdown including the closing of national borders and the suspension of immutable human rights such as the right to freedom of movement, the right to work, the right to freedom of assembly, and the right to asylum.

In many cases, executive measures were legitimised on the basis of scientific analyses. As such analyses often relied on mathematics, the understanding of mathematics had assumed a central role in governing the crisis. Thus, mathematical arguments became a part of political debates. In democratic societies, where the citizen is regarded the political sovereign and therefore needs to be able to evaluate political arguments, the question arises in how far the average citizen can be prepared by mathematics education to critically reflect on the use of mathematical arguments in political debates. We will compare the mathematics used in political debates and in the underlying scientific theories with usual contents of school mathematics and with what can reasonably be regarded as intelligible for mathematical laypersons. Due to our own cultural embeddedness, but also to limit the scope of this essay within manageable boundaries, we will confine our analysis to political debates in Germany.

Our following discussions will include an analysis of mathematical tools used in political debates on the COVID-19 crisis. To avoid any misunderstanding, we would like to underline that our analysis is not meant to evaluate the political measures taken. Instead, we will discuss in how far the presented mathematical information was suited to legitimise such measures. Note, for example, that questionable justifications can be used to legitimise executive measures which nevertheless prove efficient for other reasons.

## Evaluation of mathematical expert knowledge by citizens

In liberal democracies, the citizen is considered the sovereign power. This means that, usually through elections, citizens in a liberal democracy have the right and the obligation to reach decisions that affect society as a whole. As far as a state implemented a representative democracy, such decisions might be reached by representatives, but then again, these representatives are elected by the citizens. However, matters of political decision often build on expert knowledge, the evaluation of which might be essential to reach an informed decision. This leads to the problem that citizens, as laypersons, need to be able to critically evaluate expert knowledge in order to be able to execute their democratic rights and obligations. Hentig (1972) proposed two solutions to this problem: On the one hand, academic disciplines might want to ensure their general intelligibility. In this respect, one might think of Wille’s (2008) attempt of publicising algebra, but, all in all, general intelligibility has not become a central goal of academic research. On the other hand, it might be the task of education to prepare (future) citizens for the evaluation of expert knowledge. We will follow the latter proposal and discuss two adoptions of this proposal from mathematics education.

Skovsmose (1990) argued that “mathematics has a ‘society-shaping’ function [and] important implications for [the] development and organization of society”, and that, “[t]o make it possible to carry out democratic obligations and rights, it is necessary to be able to […] understand the functions of applications of mathematics”, including “how decisions (economical, political, …) are influenced by mathematical model building processes” (p. 111). Skovsmose (1994) warned “that the ground for decisions taken by the authorities may be inaccessible to people other than the technicians and the people in charge” (p. 39) and asked if it was “possible to secure a critical citizenship in a highly technological society” (p. 40). It might be added that even politicians would usually and especially in the current crises be laypersons and no knowledge elite at all. Skovsmose (1994) highlighted that mathematical knowledge and technological knowledge alone would not be sufficient to this end, and demanded mathematics education to strive for “reflective knowing”, which would comprise three tasks: recovering the modelled situation in its complexity, addressing problems and uncertainties in the transitions from situations to real models and mathematics models and back, and identifying in which way the mathematical model is formatting reality (pp. 97–114).

Some words seem necessary on Skovsmose’s (1994) notion of “the formatting powers of mathematics” (p. 114). Skovsmose rejected the assumption of epistemic realism that knowledge is a true and literal description of how reality really is. We share his contrasting assumption that knowledge production and validation are necessarily constructive, social, and fallible practices, also in mathematics. This is not the place for a detailed philosophical discussion of these assumptions, but it should be noted that Skovsmose’s position answers to fundamental problems of the conceptualisation and implications of epistemic realism, and is widely compatible with other theoretical frameworks implying an epistemic relativism in mathematics education, including constructivism (Thompson, 2020), socio-epistemology (Cantoral, 2020), post-structuralism, and Lacanian psychoanalysis (Walshaw, 2020). Mathematics, then, has “formatting powers” in the sense that the application of mathematics may guide (or “format”) our perception of the world we live in. A frequently discussed example of such formatting is Galilei’s (1638) modelling of the free fall with a quadratic function, despite all (then available) empirical evidence (Husserl, 1954). Porter (1995) presented a profound study of the historical development of the “role of quantitative expertise in the making of public decisions” (p. 6) and underlined that quantification in productive is creating reality and “a way of making decisions without seeming to decide” (p. 8). From this perspective, reflections on how a specific (mathematical) perspective shapes our knowing of the world, which of its aspects it puts into or fades from the spotlight, which potential and limits it brings for our understanding, become an essential part of the evaluation of expert knowledge.

Fischer (2001) worked out an educational theory in which he stressed that, in a society with a highly specialised workforce, people as laypersons will have to engage with experts. This happens in the private sphere, for example, when comparing different offers for a loan or when seeking medical counsel, and also in the public sphere, especially when citizens evaluate decisions which were reached by politicians. For Fischer, higher secondary education, especially in mathematics, should prepare for the critical evaluation of expert knowledge by laypersons:

One will usually rely on the subject-specific *correctness* of the expertise, on the fact that the expertise is up to date and that in this respect mutual monitoring between the experts of a discipline works. In the question of *significance*, i.e. how important one considers a certain expert judgement to be, how one weights it, one is dependent on one’s own judgement. In the end, you have to judge experts, even though you understand less than they do. (p. 152, original emphasis, our translation)

Fischer (2001) differentiated between three fields of knowledge in a discipline such as mathematics: *basic knowledge* about concepts and notations, *operative skills* in typical procedures, and *reflective knowledge* about mathematics. Note that Fischer’s notion of reflective knowledge did not so much mirror the general epistemic concerns of Skovsmose (1994). Rather, Fischer’s (2001) concept of reflexion was concerned with the questions “[w]hat is the meaning of the concepts and methods, what do they contribute, where are their limits” (p. 154, our translation). Fischer continued that laypersons, and that includes students in higher secondary education, should acquire operative skills only as far as they are needed for reflective knowledge. Especially for mathematics education, Fischer demanded that teaching switched its focus from learning mathematical skills to learning to reflect on mathematics.

Already Skovsmose’s (1994) wider concept of “mathemacy” explicitly assumed that reflection will have to rely on mathematical knowledge (p. 117). Fischer (2001) was more explicit when he wrote that reflective knowledge about the experts’ use of mathematics will require basic knowledge about concepts and notations and, to a limited extent, even operative skills. For example, it would hardly be possible to reflect on the potential and limits of a specific application of linear regression without any knowledge what linear regression is and how it works. This does not imply that only contents of school mathematics can be reflected upon. We understand both authors in proposing a change of the culture with which mathematical contents should be approached in the classroom and beyond towards a critically reflective culture. This would allow that citizens could approach mathematical contents beyond the school curricula with a similar reflective mindset and that this understanding of mathematics may allow for an evaluation of mathematical expert knowledge.

The objectives of both Fischer (2001) and Skovsmose (1994) resonate with studies on mathematical literacy, which “have in common that they stress awareness of the usefulness of and the ability to use mathematics in a range of different areas as an important goal of mathematics education”, thereby being “associated with education for the general public rather than with specialized academic training” (Niss & Jablonka, 2020, p. 549). In this sense, Skovsmose’s (1994) concept of “mathemacy”, which he reflected upon extensively, also building on the discussions presented above, has been acknowledged as a form of mathematical literacy (Niss & Jablonka, 2020). However, as already Apple (1992) pointed out, “mathematical literacy” is “a slippery term”, expressing rather “different agendas”, only a few of which could be characterised as a form of “critical literacy”, which is in question here (p. 423). Notwithstanding the relevance of this essay for the research field on mathematical literacy, we found it more productive to concentrate on the particular ideas presented by Fischer (2001) and Skovsmose (1994) rather than to employ a more general but less focussed perspective on mathematical literacy.

Fischer’s (2001) and Skovsmose’s (1994) approaches, maybe even any educational solution to the problem of the evaluation of mathematical expert knowledge for democratic decision making, stand and fall with the assumption that the relevant mathematical contents are intelligible to the mathematical layperson to an extent that allows for reflection. Which mathematical contents turn out to be “relevant” depends on which mathematical theories become applied in socio-political discourses and is impossible to list in advance in a conclusive form. We find this overall assumption problematic and will put it to the test by examining the mathematics behind chosen mathematical concepts that were used for public communication about the political measures enacted in reaction to COVID-19. For simplification, we will base our discussion on the construct of a “mathematical layperson” as a person who knows the mathematical contents of higher secondary education, has the corresponding mathematical skills but nothing beyond, and has either no possibility or no intention to learn all the mathematics needed to understand specific applications of higher mathematics. Thus, the main research question of this essay is: *In how far can mathematical laypersons master the mathematical knowledge necessary to evaluate political debates on COVID-19 policy?*

We will engage with political debates on COVID-19 through their presentation in German mass media. Indeed, in addition to Hentig’s (1972) two solutions, one might see mass media as a mediator between expert knowledge and layperson citizens. However, although providing readers with expert knowledge that allows for the evaluation of political decisions might be an ideal of good journalism, media are always either controlled by the state or dependent on selling their stories. Either way, their work follows a rationale that might lie at odds with any ideal of good journalism. One good example for the problems of mass media in mediating expert knowledge is media reports on anthropogenic global warming: After his examination of around 350 articles from six countries, Painter (2013) concluded that nearly 80% of the articles stressed the uncertainty of the findings, while only 15% of the articles elaborated on the epistemic nature of that uncertainty. Boykoff (2008) argued that this understanding of uncertainty led media in the USA to invite academically less influential positions on anthropogenic global warming into the debate in an attempt of balanced reporting, rather than discussion the unavoidability of uncertainty in empirical research. This practice “has served to amplify a minority view that human’s role in climate change is debated or negligent, and has concurrently engendered an appearance of increased uncertainty regarding anthropogenic climate science”, which then “permeates climate policy discourse and is used in policy decision-making” (p. 8).

Another more specific example, which has already been discussed from the perspective of mathematics education research by Greer (2009), is the estimation of Iraqi casualties of the US-led invasion of Iraq in 2003. Greer was mostly concerned with how the estimates from methodologically diverse studies were used and presented by politicians and mass media. In harmony with the educational position discussed above, Greer assumed that “an informed citizenship is essential to democratic functioning” and explained:

Although I have some knowledge of statistics, I do not claim to be competent to judge the methodological and statistical complexities of the three surveys, which raises a crucial point relevant to this paper. What are the implications for the concerned but not statistically expert citizen, faced with such apparently contradictory results from scientifically carried out surveys? (p. 107)

Greer (2009) concluded that politicians and mass media failed in sufficiently contextualising the different estimates, that reflections on the statistical methods used could only be conducted by statistical experts, but that mathematics education should prepare citizens to understand and reflect on basic statistical concepts, such as sampling and confidence intervals.

Nevertheless, there are examples of commendable science reporting for both topics, and there have been efforts to improve science reporting since the negative effects of scientifically naïve reporting on anthropogenic climate change became obvious (e.g., Schneider, 2010). In analogy to the reporting on the two topics discussed above, we are interested how mathematical aspects of the debates on pandemic policy allow for an evaluation of expert knowledge. Thus, the secondary research question of this essay is: *In how far do mass media exhibit and foster a reflective mindset towards the mathematical aspects of political debates on COVID-19 policy?*

## Attempts to understand the mathematics of the crisis

In this section, we will discuss the mathematics behind public debates on the lethality of COVID-19, the quantification of COVID-19 casualties, the reproduction number R, and the growth patterns of infection numbers. This choice represents the most prominent examples for the use of mathematical concepts in debates on pandemic policy in Germany.

### Lethality

The extent to which we are personally worried and to which political stakeholders see a need for special executive measures depends on the perception of the dangerousness of the virus. Initially, the restrictions on public life were not legitimised on the basis of the number of COVID-19 casualties but on the basis of the number of people who tested positive. However, this is a problematic number, for the more people we test, the more infected we find. There are various viruses whose rate of infection of the population is 100%. So, the infection rate alone is not the problem.

The key question is: Is it really problematic if a lot of people are infected with SARS-CoV-2? A high number of infected people are problematic when it results in serious illness, in an overload of the medical infrastructure and in human casualties. So, it is crucial to ask for the rate of infected people who need serious medical attention and for the mortality rate. We discuss these questions here only on the basis of the mortality rate, which is the proportion of people who died from COVID-19 among those who were infected with SARS-CoV-2. If we knew this rate, it would be possible to determine the expected institutional stress and the expected casualties on the basis of the number of infections and vice versa. So far, the only mathematics we need is from early secondary school, namely proportions or fractions. This leaves the impression of facing an easy mathematical problem, but the numerator and denominator that constitute our ratio are not given.

According to an early report by the World Health Organisation (2020), 3.5% of those infected had died during the first outbreak in China. The mortality rate was 5.8% in Wuhan, the city in China where the virus was first identified, but only 0.7% in the rest of China (p. 12). At that time, it was already clear that the high rate in Wuhan was associated to low test capacities which resulted in testing to be confined to severe cases. Nevertheless, the figure of 3.5% became the focus of public debate. For example, the *Süddeutsche Zeitung*, one of the largest daily newspapers in Germany, printed a warning of the high mortality with an explicit reference to the 3.5% in China (Endt et al., 2020). Also *Der Spiegel*, Europe’s largest weekly news magazine, explained that “the authorities estimate the mortality rate at around two to four percent” (Dandan et al., 2020, p. 16, our translation). On the basis of such figures, Karl Lauterbach, member of the German parliament, health economist, and the most prominent health expert of the Social Democratic Party of Germany, stated that “more than one million people could die in Germany alone” (Hammerstein & Feldenkirchen, 2020, p. 42, our translation).

The general problem here is that two widely unrelated concepts are mixed up in public communication. On the one hand, there is the assumption that a fixed proportion of infected people will die from the virus. Epidemiologists call this proportion the *lethality* of the virus. However, we neither know how many people are infected nor do we know how many infected die, as many cases of SARS-CoV-2 infections and probably also some cases of COVID-19 casualties go unnoticed. So, although lethality is an easy and very general concept, it is nearly impossible to determine directly and can only be estimated by complex models. On the other hand, it is possible to statistically determine what epidemiologists call the *case fatality rate*, that is the ratio of people who died from COVID-19 among those who tested positive for SARS-CoV-2. However, the case fatality rate in a certain environment depends heavily on the numbers of tests administered, on the sensitivity and specificity of the test, on the medical capacities to prevent severe cases from dying, and on other factors. Consequently, the case fatality rate varies strongly between different environments, and its relation to the lethality of the virus remains unclear. However, the case fatality rates of different hot spots of the crisis were the information that was easily available, and confusion with lethality seemed to have contributed to media reports that were more alarming than justified by the data.

Around the time when major events were banned in Germany, the first studies were published which estimated the lethality of SARS-CoV-2. In these studies, rates of 0.12 to 0.5% were reported (Mizumoto et al., 2020; Russell et al., 2020). It was thus established that, compared to the case fatality rates reported earlier, lethality was much lower than the value initially communicated.^1^

When we look at the mathematics used in these two studies, we find that they build on disputable assumptions and that they use mathematical techniques that require advanced tertiary studies in statistics. For example, the calculation of the lethality (there called “infection fatality ratio”) by Mizumoto et al. (2020) relies on integral equations, gamma distributions, binominal sampling, Monte Carlo Markov chains, and other mathematical tools, some of which are only referred to by reference to further publications. We assume that such approaches are unavoidable to estimate lethality as a value whose computation cannot be based on easily accessible numbers. However, this complexity of the estimation of the lethality of SARS-CoV-2 implies that any evaluation of the question in how far the applied mathematical approaches lead to reliable and meaningful estimations lies beyond the scope of the mathematical layperson. This means that laypersons cannot reflect on the mathematical theories and models in use and evaluate their formatting role for our perception of the lethality of SARS-CoV-2 in Skovsmose’s (1994) sense, nor can laypersons engage in a conceptually and critically informed conversation with experts in the sense of Fischer (2001) without an intense preparatory study of advanced mathematics. In addition, it became obvious that leading German media did not foster a reflective mindset towards the mathematics behind the reported figures but copied them uncritically. On the other hand, even laypersons can ask themselves whether the proportion of those who have to die among the infected can be a fixed number at all. They can see that we do not really know the number of infected people nor the number of people who died from the virus and that therefore any statement about lethality can only be an estimate. Likewise, the layperson can see that the death rate is biased by the uncertainties described above and also gives a problematic reflection of reality.

### The number of COVID-19 casualties

A naïve understanding of the concept of a COVID-19 casualty might be the following: Someone comes to the hospital with symptoms of a cold, tests positive for SARS-CoV-2, is treated for a few days, and then dies and counts as a COVID-19 casualty. The statistical practice, at least in Germany, indeed works like that (Schilling et al., 2020). But also, someone who visits a hospital for a different reason and gets infected with SARS-CoV-2 there eventually counts as a COVID-19 casualty. And it is also possible that a SARS-CoV-2 test is administered on a dead person, and, even then, this person might come to count as a COVID-19 casualty.

It is a complex question in how far COVID-19 is responsible for these casualties. Often, COVID-19 casualties had not only a SARS-CoV-2 infection but also underlying medical conditions, without which these people might not have died. Are these people actually casualties that have to be counted for their underlying conditions and not for COVID-19? That’s splitting hairs, of course. But at the moment when, within political decisions, the number of COVID-19 casualties decides on the freedom, health, and economic existence of millions, this hair-splitting becomes a vital distinction.

More, though not total, clarity can be obtained by an autopsy. In Germany, there is only one federal state, Hamburg, where nearly everyone who died with SARS-CoV-2 was examined for their cause of death. Wichmann et al. (2020) reported on the cause of death of the first twelve consecutive deaths with identified SARS-CoV-2 infections and found that all these patients had severe pre-existing conditions which in most cases were the main cause of death. In a press conference in May 2020, the involved researchers reported of yet unpublished data from now 192 autopsies, which again were *all* reported to have had serious medical preconditions, sometimes without awareness of them (Betzholz, 2020). From such a perspective, the number of people who died not only *with* but *because of* COVID-19 becomes impossible to determine.

The use of different models might be a solution to the problem. A simple idea might be to count one-fifth COVID-19 casualty if somebody died with five diseases that contributed to that person’s death. The total number of COVID-19 casualties would then be a cumulation of many different fractions. This might give a better estimate of the danger of COVID-19. However, such a model would lead to new difficulties, for example, concerning the identification of the appropriate fraction in each case or concerning the interpretation of the resulting numbers.

The number of COVID-19 casualties appears to be a prime example of the formatting power of mathematics as described by Skovsmose (1994). Mathematically literate laypeople should realise that a COVID-19 casualty in statistics is not necessarily someone who died from COVID-19. In the sense of Fischer’s (2001) approach, mathematically educated laypeople would have to question the experts as to why they decided to count every dead person infected with SARS-CoV-2 as a COVID-19 casualty. They would have to consider alternative ways of counting, and they would have to consider the limits of the validity of the chosen method of counting when evaluating measures.

### R

When evaluating the current trend of the epidemic, experts rely on biometric numbers. The *Berliner Morgenpost*, one of Berlin’s largest daily newspapers, reported on the use of R, the reproduction number, which they presented as the number specifying “how many people a coronavirus-infected person infects on average” (“Die Bedeutung der Reproduktionszahl”, 2020, our translation). The newspaper article further explained:

The R-value provides orientation for political decisions. When virologists measure the ‘fever’ of the pandemic, they do so according to the R-value. ‘Even if we assume that everyone contaminates 1.1 persons, we would have reached the performance limit of our health care system with the assumed intensive care beds in October’, explained Federal Chancellor Angela Merkel […] in mid-April. (our translation)

As the concept of R, as defined by the newspaper cited above, only requires an understanding of the arithmetic mean, it appears as an easy concept at first sight. However, we again face serious problems determining R. Not only do we not know how many people are indeed infected, we also get the data of identified cases with a delay of some days. The Robert Koch Institute (RKI), a German governmental agency for research on infectious diseases, therefore used a complex procedure for the daily up-to-date estimation of R.

The RKI employees Heiden and Hamouda (2020) published information on the assumptions and procedures of the calculation of R. Every infection identified in Germany had to be reported to the RKI, together with the information when the first symptoms were experienced in each case. In order to use all reported cases in the further calculation, the RKI “assigned an artificial onset of disease” if no information about the time of the first symptoms was given in the reported data (p. 11, our translation). To achieve that assignment, “a so-called multiple imputation was carried out, in which the missing data values are estimated on the basis of the statistical relationships of the known data” (ibid., our translation). For further detail, the authors referred to Little and Rubin (2020), a textbook that provides technical explanations and relies on knowledge that is well beyond introductory lectures in statistics in tertiary education. For example, Little and Rubin’s (2020) method of multiple imputation relies on an understanding of statistics with vectors, specific models of “missingness” of values, single imputation, regression models, and an abstract theory of statistical estimates. The merely technical description of the method spans over four pages with more than 20 complex formulas, depends on references to further chapters and other publications, and does not provide a justification for the statistical method (pp. 96–100).

On top of that, RKI used a procedure called “nowcasting” to estimate the number of cases that had not yet been but would be diagnosed and reported. Here, the authors referenced a biometric study published in 2014 and a publication in statistics describing a general procedure for dealing with reporting delays. As in the case of the assignment of artificial onset of disease, the concrete adaptation is not reported, neither by Heiden and Hamouda (2020) nor elsewhere. Eventually, R is computed by dividing the cumulated numbers of new infections in the last 4 days by the cumulated numbers of new infections in 4 days before that period.

As in the case of the mortality rate, the resulting values for R face a serious problem. In the public discourse, R was expected to change with the implementation of anti-spreading measures and was seen as an indicator of the success of such measures and of a de-escalating social behaviour in general. However, as the estimation of R rests not on the number of *all infected* but on the number of the *positively tested* only, the number of tests administered impacts R heavily. If the number of infections increased and the number of tests stayed constant, then this increase would be underestimated by R. If the number of tests was increased and the number of infections stayed constant, R would rise, indicating a faster spread of the virus. Heiden and Hamouda (2020) were aware of this effect:

This structural effect and the resulting increase in the number of reports can lead to the current R-value slightly overestimating the real events. An adjustment for the higher test rates is not possible without further ado, since no sufficiently differentiated test data is available. (p. 15)

Eventually, it was unclear in how far the communicated values for R were generally over- or underestimated, and how the interpretation of R should have changed with the increase of test capacity during the crisis.

In the case of R, the problem of the democratic evaluation of mathematical expert knowledge becomes very obvious. On the one hand, R constitutes a figure that has formatted our perception of the status of the pandemic, that has justified specific political measures, and that has thus made an impact on the lives of many. On the other hand, the mathematics behind R is out of reach for the general public. Thereby, to use Fischer’s (2001) distinction, the problem is not so much that the layperson lacks the operative skills to check the calculations. This can indeed be checked and delegated to experts or even to computers. The problem is that, for many of the used mathematical concepts, there is neither basic knowledge about the concepts and notations nor reflective knowledge. This makes it impossible for laypersons to recognise these concepts, to understand what they are for, or to even reflect on the potential and limitations of these concepts in relation to the field in which they are applied. This is how socially influential uses of mathematics slide out of democratic control.

### Exponential growth of infections

German media, experts, and politicians repeatedly warned that infection numbers in Germany would rise exponentially if no special measures were taken (e.g., Heiden & Hamouda, 2020; Müller-Jung, 2020). This idea relies on a mathematical model that is taught in German secondary schools and assumes that every infected person infects a fixed number of other persons. Exponential growth was often used to legitimise warnings against the threat posed by SARS-CoV-2. For example, the German popular mathematician Beutelspacher explained that “this growth is usually so that you don’t notice anything at all in the beginning and you are tempted to underestimate it all [but] once the momentum starts, it’s almost unstoppable”, only to add that “This is a growth that people do not understand” (Welty, 2020). But was it sound to expect that mysterious growth?

**Fig. 1:**
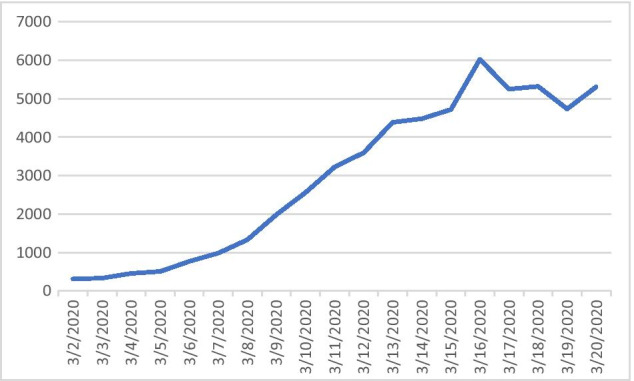
Number of reported and estimated daily onsets of COVID-19 in Germany (Robert-Koch-Institut, 2020)

Figure 1 shows the number of reported and estimated daily onsets of COVID-19 for the period before the lockdown started in Germany.^2^ Following the exponential growth assumption, the number of daily onsets should have grown proportionally to the number of overall infections, which appears not to be the case. Indeed, regression analysis proposes that a linear model (${R}^{2}\approx .960)$ is much more appropriate than an exponential model (${R}^{2}\approx .417)$.

The exponential growth assumption was problematic also from a mathematical perspective. One problem is that infection numbers cannot grow without limit. As the number of human beings is limited, so are infection numbers. The more people are infected with SARS-CoV-2, the more difficult it gets for an infected person to find a non-infected person to infect. This phenomenon of saturation can be modelled mathematically and leads to growth scenarios which are approximately exponential only in the very beginning. Thereby, we do not even know if the saturation rate of SARS-CoV-2 would lie at 100% or lower. For example, it could be that previous infections with SARS-CoV-2 or a sufficiently similar coronavirus caused an immunity against SARS-CoV-2 among a considerable proportion of the population. Furthermore, increasing test capacities might lead to an increase in case numbers without saying anything about the increase of the number of infections. Another problem is that exponential growth assumes constant doubling times, while we witnessed strong fluctuations in the doubling times of reported new cases even before the first lockdown. It appears that people already became more careful when they consumed media reports on the danger of COVID-19.

Exponential growth also provides an example of how mathematics does not represent reality but formats it in Skovsmose’s (1994) sense. The exponential model appeals through its simplicity and through the beauty of its pattern. Against this attraction, reality can apparently only with difficulty assert itself. The layman’s task is to ascertain from the expert what speaks for and against the assumption of an exponential development. However, the laypersons’ task as seen in Fischer’s (2001) approach could also be reversed: They may assume the responsibility for short circuiting the real developments with the mathematical model and, if necessary, for prompting the expert to revise his modelling.

## Reflections of our attempts

We raised the research questions in how far mathematical laypersons can master the mathematical knowledge necessary to evaluate political debates on COVID-19 policy and in how far mass media exhibit and foster a reflective mindset towards the mathematical aspects of political debates on COVID-19 policy. The first question included the more specific question whether school mathematics would be sufficient for such understanding or at least a realistic basis to build up the necessary mathematical understanding.

We have selected four discourses that have emerged around COVID-19 for our analysis. In the four discourses, we faced concepts whose definitions rested on mathematics that was well within usual secondary school curricula, at least in Germany. Proportions allowed to understand the idea of lethality; the number of COVID-19 casualties appeared to be a case of mere addition; the reproduction number can be understood as the arithmetic mean of a factor; the development of infection cases could be understood on the grounds of exponential functions. Despite the fact that these mathematical models were presented in such simplicity by mass media, they all turned out to be problematic. In the case of the exponential function, which we will address later, the model used appears to be simply misleading.

In the cases of lethality, the number of COVID-19 casualties, and the reproduction number, we faced ideal concepts, which are easy to define and possible to understand as mathematical laypersons but utterly impractical or even impossible to actually determine. In each case, the numbers that were determined and used in the public discourse were something rather different than the ideal concepts originally laid out. In the case of lethality, we saw that our limited data yields inappropriately high rates, is built on contestable modelling assumptions, and uses mathematical techniques that lie beyond the contents of a usual degree course in mathematics and statistics. The same is true for the reproduction number, whose significance remained questionable. In the case of the count of COVID-19 casualties, the mathematics was well within the contents of school mathematics, but the modelling assumptions were highly problematic. It should be noted that we did not discuss studies based on excess mortality rates as alternative statistical approaches towards estimations of COVID-19 casualties (e.g., Stang et al., 2020). Such approaches have the potential to overcome the modelling flaws of the counting method described here, but then, they are again based on mathematics that is well beyond the contents of secondary school mathematics. A first result from this analysis is that initial mathematical understanding of the presented discourses is possible but misleading, as this initial understanding concerns only ideal concepts, which cannot be determined empirically. In contrast to that, a profound understanding of what is happening mathematically is out of reach for mathematical laypersons. This insight mirrors Greer’s (2009) statement that “[t]he technical aspects of the methodology [of the estimation of Iraqi casualties] are complex to the point that even a mathematician without specialized knowledge is not well placed to evaluate the results” (p. 112).

Obstacles towards the evaluation of mathematical expert knowledge in debates on COVID-19 policy might be less problematic if a critical analysis of the mathematics motivated no criticism. The case of exponential spread models for SARS-CoV-2 shows that such serenity would be far from appropriate. As we could see, the publicly communicated danger of an exponential increase of SARS-CoV-2 infections is neither mathematically sound nor empirically valid. From a mathematical perspective, the narrative of an exponential increase might again be understood as a simplification for the sake of public communication. Indeed, mathematical models for the spread of infectious deceases assume a spread behaviour which is almost exponential in the early phase of an epidemic. However, empirical data of the number of SARS-CoV-2 infections did not provide any reason to assume an exponential increase, as even laypersons could conclude from a view on the respective graphs.^3^ Our interpretation is that we witness mathematics as a formatting power here. On the one hand, the mathematical models allowing a prediction are too complex for laypersons and of yet unverified applicability. On the other hand, the public just as most journalists, politicians, virologists, epidemiologists and other mathematical laypersons know the exponential function, which allows an easy explanation of the development of infection numbers. We propose to consider whether it was the availability alone of the exponential function as a description of increasing numbers of infections that caused its use. If this was the case, then we would face a case of the formatting of reality on the basis of the mathematics available: Rather than using mathematics for a reasonable *description* of empirical data, the mathematical concept of exponential growth is used to *format* our expectations of the development of infection numbers. It might not be a coincidence that this formatting allows for much more alarming predictions than other models.

Although it may be argued that mathematics education can prepare students to reflect on applications of higher mathematics (e.g., Greer, 2009), we face the paradox situation that mathematics education is a condition of possibility of the reported problems: Only because the citizen has been enculturated into basic mathematical concepts in the mathematics classroom, it is possible to use such understanding for the ideal definitions of the mathematical concepts, thus creating the confusion between ideal concepts and mathematically complex estimations in the political debates on COVID-19 policy in the first place. From a critical perspective, this implies that mathematics education cannot be seen as separate from the use of mathematics in political debates, that it is always-already political. The question then is if mathematics education should ignore the mingling of ideal and empirical mathematical concepts, or if it should allow for the critical reflection of such applications of mathematics.

Concerning the question in how far mass media exhibit and foster a reflective mindset towards the mathematical aspects of political debates on COVID-19 policy, we have to give a pessimistic, though somewhat nuanced, answer. As we outlined above, most newspaper reports were effective in creating the problems that we addressed: They presented the discussed mathematical concepts in their ideal forms but then gave estimations of the respective figures without discussing the differences between the two conceptions or the assumptions and methods behind the reported figures. This suggests that mass media still fail to present scientific models and results in a way that allows for mathematical reflection and a critical evaluation of such information by citizens. However, there have also been notable attempts towards a more reflected discussion of the respective mathematical concepts, as, for example, in the following two examples. Ironically, such attempts often build their arguments on the debated figures in spite of their general scepticism. One example is Müller-Jung’s (2020) article in the *Frankfurter Allgemeine*, Germany’s third largest daily newspaper. Müller-Jung first stated:

The lethality or mortality rate is also not easy to determine because a large proportion of infected persons have not yet been identified. If the mortality rate is low and the infection is mild or asymptomatic, many virus carriers do not appear at all in clinics, at doctors and thus in any statistics. (our translation)

Thereafter, the only numbers he presented to answer the question “How deadly is the virus?” are those of the “case-related lethality” which would be estimated “between 2 and 4 percent” (our translation). Another example is Weimer’s (2020) article in *The European*, a German-English political magazine, where Weimer started with an estimation of 1.9 million casualties in Germany based on an expected lethality of 3.4%, only to discuss afterwards that such a high number was rather unlikely and due to insufficient data. It should be noted that both articles did not present alternative data, on the basis of which less alarming predictions could be made. Both articles clearly explicated why using the current numbers does not make much sense. In spite of this insight, they could not refrain from using these numbers for their arguments.

We want to finish our reflections with a possible explanation of the reported use of mathematics, which is not addressed as often as it possibly should be, given its explanatory power. Early on, we reported that Skovsmose (1994) rejected epistemic realism and assumed a position of epistemic relativism. Notice the implications of both positions for the presentation of mathematical concepts:

- From a relativist perspective, knowledge may be useful, but it is never literally true. Instead, it is mind-dependent, allows for a particular perception of the real, and is necessarily factionary. Thus, the lethality of COVID-19 and the number of COVID-19 casualties are mind-made concepts, whose meaning is formed by our use. Therefore, every application of mathematics should go hand in hand with a discussion of its potential and its limitations in building an understanding of our world.
- From a realist perspective, however, knowledge is a literal and true description of reality. Thus, the lethality of COVID-19 and the number of COVID-19 casualties exist independently of our awareness of them. Models to calculate such figures do not create them in the first place, but lead to ever better approximations of the true figures that wait “out there” for discovery. The quality of such approximations is merely a technical detail but does not call into question the significance of the mathematical concept.

Such realist positions have been attacked for obscuring how mathematics is formative in our understanding of the world in ways that might call for public evaluation. Obviously, the media reporting of the mathematics involved in the debates on COVID-19 policy, problematised here from a relativist perspective, appears perfectly natural from a positivist position. This suggests that many journalists hold a realist mindset concerning mathematics. Such a mindset, and the resulting deficits in an epistemically reflective reporting of applications of mathematics, can be seen as direct results of mathematics education in schools, given that mathematics education has been repeatedly documented to foster naïve realism (e.g., Dowling, 1998; Ullmann, 2008).

## Conclusions

On the basis of the reflections of our attempts to understand the mathematics behind the selected discourses on the SARS-CoV-2 crisis, we notice that we face highly relevant and controversial political debates, whose mathematical components cannot be understood by mathematical laypersons to an extent that would allow for the evaluation of such expert knowledge. In addition, media reports usually do not facilitate such an evaluation and often provide problematic accounts of the respective mathematical concepts. Thus, journalism mirrors the limitations of mathematical laypersons and does not appear to systematically invest into a more profound reflection of the mathematics involved.

This does not mean that the idea of the evaluation of expert knowledge by citizens failed in general. First, in negotiations of a private loan or when discussing which taxes to raise how drastically for the funding of a certain welfare policy, the underlying mathematics might be familiar from secondary education or at least approachable for the mathematical layperson. Second, Fischer’s (2001) distinction between basic knowledge, operative knowledge, and reflective knowledge about mathematics allows us to ask in how far laypersons can build up reflective knowledge or be provided such knowledge by media, as at least indicated in a few news reports such as the ones by Müller-Jung (2020) and Weimer (2020). Here, it remains an open question in how far mathematical concepts and models can be reflected upon without any detailed knowledge of the concepts, notations, and operations involved. Therefore, on the one hand, we still support Skovsmose’s (1994) and Fischer’s (2001) assumption that reflections on mathematics can allow for the evaluation of mathematical expert knowledge and must play a central role in mathematics education. On the other hand, we discern that critical reflection of mathematics cannot be the sole solution to the problem it stood up against, for too often the mathematics in question is out of reach of the mathematical layperson.

This insight leads to the question if and how the evaluation of higher mathematics for the citizen’s decision-making is possible. In his discussion of estimates of Iraqi war casualties, Greer (2009) argued that “it would be helpful to have a group of ‘honest brokers’ to mediate between the ‘constructors’, ‘operators’, and ‘consumers’ of the mathematical formulations that play such an, often invisible, role in formatting our perceptions of social issues” and added that “[t]his is a role that mainstream media conspicuously fail to play” (p. 110). It might be a solution to institutionalise mathematical experts with the task to facilitate the evaluation of socially relevant applications of mathematics for laypersons, but, then, such a solution could create new experts whose evaluation would have to be organised. It might be a consequence of this iteration that such evaluation cannot be institutionalised but has to take place in a decentralised manner. A promising conceptualisation of the latter idea can be found in Hauge and Barwell’s (2017) discussion of “extended peer communities”. They cited Funtowicz and Ravetz (1993), who stated that, “with mutual respect among various perspectives and forms of knowing, there is a possibility for the development of a genuine and effective democratic element in the life of science” (p. 741). However, at least in Germany, the debates on COVID-19 policy suggest that such “mutual respect” is not yet within sight, given that, to provide only one example which is symptomatic for the overall atmosphere during the first lockdown, e German chancellor Merkel criticised “discussion orgies”, when the respective discussions questioned the appropriateness of political decisions (Chornley, 2020).

We conclude that mathematics education is inseparably involved in the problematic use of mathematics in political debates, that it is implicitly preparing such problematic use by creating the condition of its possibility and by relying on problematic epistemic assumptions, and that it has the potential to prepare a more reflected position towards applications of mathematics. Notwithstanding that the choice of curricular contents necessary for this endeavour might require regular revision, it is obvious that mathematics education lays the basis for the reflection of the use of mathematical models by experts. As demanded by Skovsmose (1994) and Fischer (2001), the respective discussions of mathematical models should include reflections of the potential and limits of the application of specific mathematical contents in addition to conceptual and procedural knowledge. However, the further the mathematics used lies beyond the scope of the mathematical layperson, the more will the evaluation of expert knowledge have to be based on a more general sceptical mindset towards the application of mathematics and on the commitment of extended peer communities. While Skovsmose (1994) and Hauge and Barwell (2017) presented possible trajectories to that end, it might still be a long way to go before the mathematics classroom will contribute to the mathematical education of the citizen to the outlined extent.

## Footnotes

1 This lethality is in the range of conventional influenza viruses. However, lethality alone cannot describe the dangerousness of SARS-CoV-2, as it does not take into account, for example, which medical measures are required to save a patient, the speed of the spread of the virus, or possible long-term effects on patients who had endured an infection.

2 It should be noted that from 9 March 2020 on, events with more than 1000 participants were forbidden, and from 16 March 2020 on, further regulations were implemented. Assuming an incubation period of 4 days, it should be reasonable to present data up to 20 March 2020. In the weeks after that date, the graph was more or less constant.

3 One of the authors of this article was contacted by a layperson who had made the observation that the graphs are rather linear in most federal states of Germany and other countries.
